# Postsurgical tactile-evoked pain: a role for brain-derived neurotrophic factor-tropomyosin receptor kinase B–dependent novel tactile corpuscles

**DOI:** 10.1097/PR9.0000000000001169

**Published:** 2024-08-08

**Authors:** Kirsten Wilson, Ying Sze, Anna Regan, Chunyi Zhu, Katarzyna Mazur, Atanaska N. Velichkova, Carole Torsney

**Affiliations:** a Centre for Discovery Brain Sciences, University of Edinburgh, Edinburgh, United Kingdom. Wilson is now with the School of Biological Sciences, University of Edinburgh, Edinburgh, United Kingdom. Velichkova is now with the Charles River Laboratories, Groningen, Netherlands; b Simons Initiative for the Developing Brain, University of Edinburgh, Edinburgh, United Kingdom

**Keywords:** Tactile, Dynamic allodynia, Meissner corpuscle, Post-surgical pain, Skin

## Abstract

**Introduction:**

Millions of people undergo surgical procedures each year with many developing postsurgical pain. Dynamic allodynia can arise when, for example, clothing brushing close to the surgical site elicits pain. The allodynia circuits that enable crosstalk between afferent tactile inputs and central pain circuits have been studied, but the peripheral tactile drive has not been explored.

**Objective:**

Investigate the innervation of the skin in the rat plantar hindpaw skin–muscle incision model.

**Results:**

Incision increased epidermal thickness and cell layers and reduced intraepidermal nerve fibre density, identified with PGP9.5 immunostaining. Strikingly, Collagen IV immunostaining revealed the development of dermal protrusions, oriented towards the incision site, that were reminiscent of the dermal papillae that exist in glabrous footpads. S100 immunostaining for lamellar Schwann cells revealed the presence of novel tactile corpuscles (S100-positive bulb) within incision-induced putative dermal papillae. The occurrence of these novel tactile corpuscles coincided with behavioural observations of dynamic allodynia. Tactile corpuscles require brain-derived neurotrophic factor- tropomyosin receptor kinase B (BDNF-TrkB) signalling to form during development, and an increase in BDNF-immunostaining intensity was observed close to the incision site. Local acute administration of TrkB-Fc, to block BDNF-TrkB signalling, reduced, by approximately 50%, both tactile corpuscle size (S100+ bulb area) and dynamic allodynia.

**Conclusion:**

Surgery induces the development of novel tactile corpuscles in the incision surround, in a BDNF-TrKB–dependent manner, that contributes to postsurgical tactile-evoked pain.

## Abstract 2

Surgery induces the development of novel tactile corpuscles in the incision surround, in a brain-derived neurotrophic factor-TrKB–dependent manner, that is key for postsurgical tactile-evoked pain.

## 1. Introduction

Worldwide, millions of patients undergo surgery each year^51^ with 30% to 80% developing moderate to severe postsurgical pain.^5,19^ This pain is poorly managed^5^ due to a limited understanding of the underlying mechanisms.^13,18^ This can lead to persistent postsurgical pain following routine procedures^16,28^ and a risk of opioid misuse.^8^ A common yet disturbing symptom is “dynamic allodynia,” when tactile stimuli such as clothing close to the surgical site elicits pain sensation. This is observed following multiple types of surgery, for example, breast,^17,23^ thoracic,^20^ abdominal,^1,2,52^ lower limb,^36,41^ foot,^26^ and hand,^40,43,44^ and can last for months^17,20,36,41,43,44^ and even years.^1,2^

The rodent hindpaw plantar (skin-muscle) incision model developed by Brennan et al. in 1996 has facilitated research into the mechanisms of postsurgical pain.^9^ Study of this and subsequent related preclinical surgical models provides evidence for both inflammatory and neuropathic components with the involvement of peripheral sensitisation, central neural plasticity, and neuroimmune interactions.^13,18,28^ Behavioural readouts from multiple studies report thermal hyperalgesia and punctate mechanical hypersensitivity (reviewed).^13^ However, more closely aligned with the patient experience is the observation of dynamic brush allodynia in the mouse hindpaw plantar incision model^39^ that we also report here in the rat.

Mechanical allodynia involves crosstalk between tactile inputs and pain circuits in the spinal cord. These “spinal allodynia” circuits comprise overlapping yet distinct circuits for inflammatory vs neuropathic allodynia with specific circuits for dynamic vs punctate mechanical allodynia.^38^ There has been some investigation of the tactile afferents that provide input to these spinal allodynia circuits. TrkB-expressing primary sensory neurons have been shown to be required for neuropathic but not inflammatory pain.^14^ Interestingly, following nerve injury, an abnormal skin reinnervation dominated by large diameter primary sensory neurons, that likely includes tactile afferents, is concurrent with neuropathic allodynia.^29^ Moreover, abnormal reinnervation of tactile end organs can drive neuropathic pain.^21^ However, the impact of surgical incision upon local skin innervation has not been explored, and the tactile inputs that drive postsurgical tactile-evoked pain have not been defined.

## 2. Methods

### 2.1. Animals

All experiments were approved by the University of Edinburgh Ethical Review Committee and carried out in accordance with the UK Animals (Scientific Procedures) Act 1986. Adult male Sprague-Dawley rats, 6 to 8 weeks of age (University of Edinburgh Bioresearch and Veterinary Services), were used (n = 57). A small cohort of adult female subjects (6–8 weeks) was also studied (n = 8). Animals were housed in cages at 21°C and 55% relative humidity, with a 12-hour light–dark cycle and food and water provided ad libitum.

### 2.2. Hindpaw incision model

Animals underwent surgical incision of the left hindpaw under isoflurane anaesthesia and aseptic conditions at approximately 6 weeks of age. As previously established,^9^ a longitudinal incision was made through the plantar aspect of the hindpaw skin and fascia from the midpoint of the heel to the first skin pad with a number 11 blade. The underlying plantaris muscle was elevated and also incised longitudinally. The wound was closed with two 3-0 silk sutures followed by the application of 3M Vetbond tissue glue. For the subset of subjects followed until postsurgical day (PSD) 7, any remaining sutures were removed under brief isoflurane-induced anaesthesia on PSD4.

### 2.3. TrkB-Fc chimera administration

To assess the involvement of local brain derived neurotrophic factor- tropomyosin receptor kinase B (BDNF-TrkB) signalling, 3 treatment groups were compared, incision only, incision + vehicle or incision + TrkB-Fc chimera. Treatments were administered immediately following surgery, when still under anaesthesia, following suturing and tissue glue application to minimise drug leakage, as previously conducted.^54^ A second administration was conducted on PSD2 under brief anaesthesia. For each administration, ×4 25 ng/12.5 µL TrkB-Fc chimera (#688-TK, R&D Systems, Minneapolis, Minnesota, USA) or vehicle (sterile phosphate-buffered saline [PBS]) injections were administered, ×2 either side of the incision site, as recommended by the University of Edinburgh Bioresearch and Veterinary Services.

### 2.4. Behavioural sensory testing

Behavioural testing was conducted before and 1–7 days after hindpaw incision. Following 3-day habituation on an elevated glass platform maintained at 30°C, radiant heat was applied to the midplantar surface of the hindpaw (3 times per hindpaw to calculate average) to determine the noxious thermal withdrawal latency using the Hargreaves apparatus (IITC Life Science). Similarly, following 3-day habituation on an elevated mesh platform within clear Perspex compartments, the mechanical threshold of the nociceptive flexion withdrawal reflex was determined using an electronic von Frey apparatus (Ugo Basile, Varese, Italy), applied to the midplantar surface of the hindpaw (3 times per hindpaw to calculate average). On the same elevated wire mesh platform within clear Perspex compartments, dynamic mechanical allodynia was measured, by applying a paintbrush (Round #2, Daler-Rowney) to the plantar surface of the paw from under the wire mesh, in a heel to toe direction for approximately one second. The response was scored: 0 = no response/walking away, 1 = single withdrawal (flick or stamp), 2 = multiple withdrawals in rapid succession, and 3 = licking of the plantar surface or continued elevation/withdrawal of the hindpaw (adapted from Duan et al. [2014]^15^). Each hindpaw was tested 3 times, and the total for each paw was calculated.

### 2.5. Skin immunostaining

Animals were anaesthetised by inhalation of isoflurane before being decapitated. Tissue from the entire plantar aspect of both hindpaws was collected and post-fixed in 4% wt/vol paraformaldehyde in 0.1M PBS solution at 4°C for 2 hours, before being transferred into 20% wt/vol sucrose in 0.1M PBS solution overnight at 4°C. Biopsies were collected from the incision site on the ipsilateral heel, as well as an equivalent control site on the contralateral heel and a contralateral control footpad on PSD1, PSD2, PSD3, and PSD7. Biopsies were OCT embedded and stored at −20°C until 30-µm cryostat sections were cut. For comparative purposes, serial sections were collected, allowing different antibody combinations to be tested on the same skin sample and ensuring that sections, for a given antibody combination, were at least 90 μm apart.

Free-floating tissue sections were incubated in 0.1M glycine solution, before being blocked in 10% normal donkey serum (NDS) in PBST (PBS containing 0.3% Triton-X-100) for 1 hour. Antibody diluent contained 10% or 5% NDS in PBST, for primary and secondary antibody incubations, respectively. Primary antibody incubation occurred overnight at room temperature with rabbit antiserum to PGP9.5 (1:500, DAKO/Agilent, Santa Clara, California, USA), goat antiserum to Collagen IV (1:500, Cambridge Bioscience, Cambridge, United Kingdom), rabbit antiserum to S100 (1:2000, Dako), mouse antiserum to Neurofilament 200 (NF200, 1:500, Sigma-Aldrich, St Louis, Missouri, USA), or rabbit antiserum to Brain Derived Neurotrophic Factor (BDNF, 1:200, Sigma-Aldrich). Secondary antibody incubation was for 4 hours with donkey anti-rabbit AlexaFluor 568 (1:750, Jackson ImmunoResearch Europe Ltd, Ely, United Kingdom), donkey anti-goat AlexaFluor 488 (1:750, Jackson ImmunoResearch), donkey anti-mouse 488 (1:750, Life Technologies, Paisley, United Kingdom), or donkey anti-goat 405 (1:250, Abcam, Cambridge, United Kingdom) before, for a subset, a final incubation in DAPI (1:33,000, Sigma Aldrich).

### 2.6. Image capture and analysis

Images for analysis were taken using a Leica DMR fluorescence microscope using ×5 (BDNF, wound area), ×10 (Collagen IV/S100/NF200), and ×20 (PGP9.5, epidermal thickness/cell layers, S100+ bulb area) magnification objectives. Images were taken of 3–4 sections per animal using Leica Application Suite software. Images were taken of the incision heel to one side of the incision site (with the “wound plug” visible), the centre of the control heel, and the centre of the control footpad: 1×10 image (1360-µm mediolateral extent); 2 ×20 images (total 1360-µm mediolateral extent); 2 ×10 images (TrkB-Fc experiment only; total 2720-µm mediolateral extent). The experimenter was blind to postsurgical day; however, it was not possible to be blind to “site” as all 3 are strikingly different. However, for analysis of incision tissue in the TrkB-Fc administration experiment, this was conducted blind to treatment group. Representative images were captured using a Nikon A1R confocal microscope system using ×4 and ×20 magnification objectives.

Epidermal “thickness” was measured as the distance between the basement membrane, visualised using Collagen IV immunostaining, to the outermost cell layer, not including the stratum corneum, which was not retained in all tissue sections. The number of epidermal cell layers was counted from the basement membrane, to the outermost cell layer in DAPI-stained sections. Both epidermal thickness and cell layer measurements were made at the midpoint of 2 ×20 images and averaged per section. PGP9.5-positive intraepidermal nerve fibres that crossed the basement membrane into the epidermis were counted and expressed per millimetre of basement membrane. If fibres branched once they crossed the basement membrane, they were counted as one fibre. Dermal papillae were classified as protrusions of the basement membrane into the epidermis that measured at least 30 µm in height. Dermal papillae were counted and expressed per millimetre of basement membrane. In addition, the height of each papilla was measured. Each papilla was examined for S100-positive Schwann cell and NF200-positive myelinated A-fibre immunostaining and expressed per mm basement membrane. A tactile (Meissner) corpuscle was defined by the presence of an S100-positive bulb (a bulbous structure with a girth visibly larger than S100+/NF200+ nerve fibres) as routinely employed.^34,35^ S100-positive bulbs were also examined for associated NF200-positive nerve fibre staining, but this was not used to define tactile corpuscles because it could underestimate the density of tactile corpuscles as the larger S100+ bulb will not always be associated with the thinner innervating nerve fibre in a given sectioning plane. The number of papillae containing an S100-positive bulb (±NF200+ immunostaining) or an S100+ bulb with NF200+ immunostaining was each counted and expressed per mm of basement membrane. S100+ bulb area was measured with a minimum threshold value of 100 µm^2^. Brain-derived neurotrophic factor immunofluorescence intensity was measured in the epidermis at the centre of the control heel, centre of the control footpad, and immediately adjacent and also distal (2300 µm) to the incision site using 3 small (1460 µm^2^) regions of interest that were averaged for each site. The averaged BDNF-immunofluorescence intensity, for each site, was normalised to control footpad values in each subject, or control heel in the TrkB-Fc experiment, to account for potential differences in immunostaining intensity between the subjects. Cross-sectional wound plug area was measured in Collagen IV immunostained sections. All measurements were made using Fiji software (version 1.54).

### 2.7. Statistical analysis

Behavioural data were analysed using repeated-measures (RM) 2-way ANOVA followed by Sidak multiple comparisons posttests, which compared ipsilateral and contralateral hindpaw values at each PSD time point. Immunohistochemical time line data were analysed using RM 2-way ANOVA followed by Sidak multiple comparisons posttests (incision heel vs control footpad) or Tukey multiple comparisons test (incision heel vs control heel vs control footpad) at each time point. Treatment group (vehicle/TrkB-Fc chimera) comparison with the incision only group was analysed using RM 2-way ANOVA followed by Sidak multiple comparisons for behavioural studies. Within-treatment group comparison was analysed using one-way ANOVA followed by Tukey multiple comparisons test for BDNF immunostaining. Treatment group (vehicle/TrkB-Fc chimera) comparison with the incision-only group for anatomical measures was analysed using one-way ANOVA followed by Dunnett multiple comparison test or Kruskal–Wallis test followed by Dunn multiple comparison depending on normality of data sets assessed using D'Agostino and Pearson test. For all statistical analysis, *P* values less than 0.05 were considered significant, and all data are presented as mean ± SEM.

## 3. Results

### 3.1. Behavioural hypersensitivity

**Figure 1.**
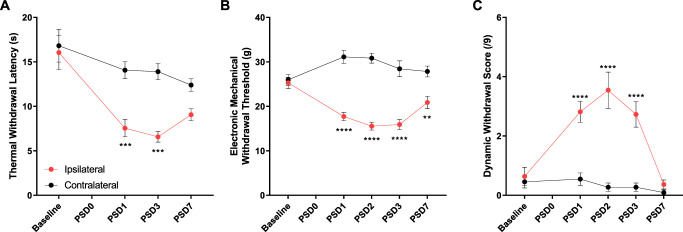
Hindpaw surgical incision results in thermal hyperalgesia, punctate mechanical allodynia, and dynamic mechanical allodynia. (A) Ipsilateral hindpaw withdrawal latencies to noxious radiant heat stimuli (Hargeaves) are reduced on PSD1 and PSD3. (B) Ipsilateral hindpaw punctate mechanical withdrawal thresholds (electronic von Frey) are decreased on PSD1, PSD2, PSD3, and PSD7. (C) Ipsilateral dynamic mechanical sensitivity (paintbrush stroking withdrawal score) is increased in the ipsilateral hindpaw on PSD1, PSD2, and PSD3. Statistical analysis: RM 2-way ANOVA, Sidak multiple comparisons posttests shown (*P* < 0.01**, *P* < 0.001***, *P* < 0.0001****). n = 6 male subjects.

Hindpaw plantar incision resulted in the development of thermal hyperalgesia evident as a reduced thermal withdrawal latency in the incised hindpaw on PSD1 and PSD3 that had resolved by PSD7 (Fig. 1A). Punctate mechanical hypersensitivity, as assessed using electronic von Frey, was also observed with reduced mechanical thresholds on PSD1-3 and PSD7 (Fig. 1B). Dynamic mechanical allodynia, measured using a paintbrush assay, was observed on PSD1-3 and had resolved by PSD7 (Fig. 1C).

### 3.2. Epidermal structure and innervation

**Figure 2.**
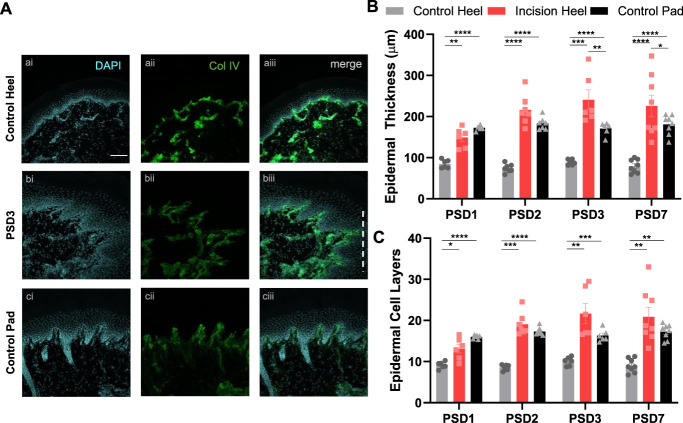
Hindpaw surgical incision increases epidermal thickness and cell layers. (A) Control heel (a), PSD3 incision heel (b), and control pad (c) with nuclear stain DAPI (cyan, i) and Collagen IV immunostaining (green, ii) and overlayed in (iii). Dashed line in (biii) marks direction of incision site. Scale bar 100 µm, all panels. (B) Hindpaw heel incision increases epidermal thickness, across all postsurgical days and exceeds that of the normally thicker control pad region on PSD3 and PSD7. (C) Hindpaw heel incision increases the number of epidermal cell layers across all postsurgical days such that it is comparable to the control pad region. RM 2-way ANOVA, Tukey multiple comparisons posttests shown (*P* < 0.05*, *P* < 0.01**, *P* < 0.001***, *P* < 0.0001****). n = 6 to 8 male subjects per PSD.

To assess gross epidermal structure, the epidermal–dermal border (basement membrane) was visualised using Collagen IV-immunostaining alongside the nuclear stain DAPI, to visualise epidermal cell layers (Fig. 2A). Epidermal thickness was significantly increased in the incision heel, compared with the control heel across all time points (Fig. 2B). A comparison was also made with the normally thicker footpad region, revealing that the incision heel epidermal thickness was significantly greater than that of the control footpad on PSD3 and PSD7. Similarly, the number of epidermal cell layers was significantly increased in the incision heel, compared with the control heel across all postsurgical days (Fig. 2C).

**Figure 3.**
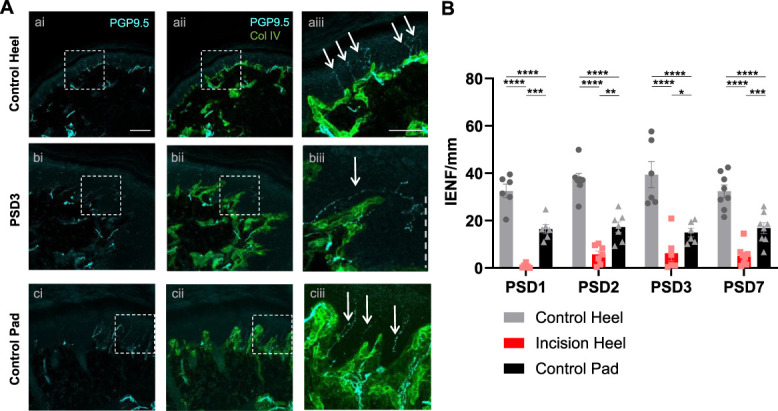
Hindpaw surgical incision decreases intraepidermal nerve fibre density (IENFD). (A) Control heel (a), PSD3 incision heel (b), and control pad (c) with PGP-9.5 (cyan, i) and Collagen IV immunostaining (green, ii) and overlayed in (iii). Dashed line in (biii) marks direction of incision site. Arrows denote PGP9.5 positive nerve fibres that cross the basement membrane into the epidermis (intraepidermal nerve fibres). Scale bar ai. 100 µm (relevant for all i. and ii. images); aiii. 50 µm (relevant for all iii. images). (B) Hindpaw heel incision decreases intraepidermal nerve fibre density (IENFD), across all postsurgical days. The control pad has a lower IENFD compared with the control heel. RM 2-way ANOVA, Tukey multiple comparisons posttests shown (*P* < 0.05*, *P* < 0.01**, *P* < 0.001***, *P* < 0.0001****). n = 6 to 8 male subjects per PSD.

Epidermal nociceptive innervation was assessed by quantifying the intraepidermal nerve fibre density (IENFD) visualised with PGP9.5-immunostaining (Fig. 3A). The density of PGP9.5-immunopositive nerve fibres crossing the basement membrane, visualised with Collagen IV immunostaining, was compared between the 3 sites (Fig. 3B). Intraepidermal nerve fibre density was highest in the control heel compared with the control footpad but was significantly reduced in the incision heel across all postsurgical days.

In summary, in the incision site surround, epidermal hyperplasia was observed accompanied by a reduction in IENFD on PSD1-7.

### 3.3. Dermal structure and innervation

**Figure 4.**
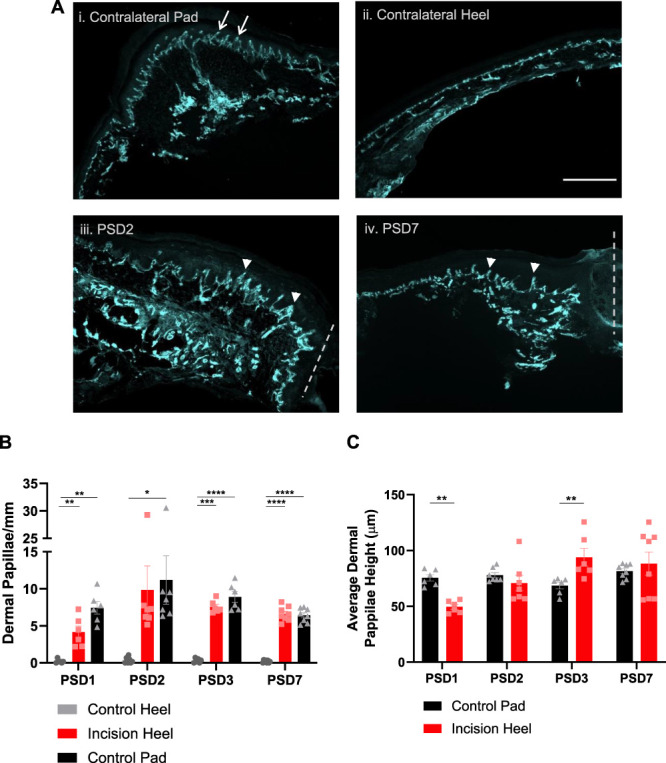
Hindpaw surgical incision induces putative dermal papillae. (A) Collagen IV immunostaining of the dermal–epidermal junction (basement membrane) enables visualisation of dermal papillae (white arrows denote examples) in control pad (i), but not control heel (ii) and reveals putative dermal papillae (arrowheads denote examples) oriented towards the incision site (dashed line iii, iv). Scale bar 500 μm (i-iv). (B) Dermal papillae density and (C) average height reveals that incision-induced putative dermal papillae peak in density and height ∼PSD2/3 and are retained at PSD7 with measures comparable to that observed in the control footpad. RM 2-way ANOVA, Tukey multiple comparisons posttests shown (*P* < 0.05*, *P* < 0.01**, *P* < 0.001***, *P* < 0.0001****). n = 6 to 8 male subjects per PSD.

The pad regions of the glabrous hindpaw skin are enriched with dermal papillae that contain tactile (Meissner) corpuscles—sensory end organs that detect fine touch.^24^ These papillae can be visualised with Collagen IV immunostaining of the epidermal–dermal junction (Fig. 4Ai). The heel region normally lacks dermal papillae (Fig. 4Aii). Strikingly, following incision in the heel region, putative dermal papillae developed close to and appear to be oriented towards the incision site (Fig. 4Aiii, iv also evident at higher magnification in Figs. 2Ab and 3Ab). The density of the incision-induced putative dermal papillae is comparable to that of dermal papillae that exist in the hindpaw pads (Fig. 4B). Moreover, the average height of the putative dermal papillae gradually increased postincision to be comparable to the control pad by PSD2, exceeded this by PSD3 but were still present and of a comparable height at PSD7, when dynamic allodynia had resolved (Fig. 4C). This led us to examine whether postincision putative dermal papillae are innervated with tactile corpuscles concurrent with the observed dynamic allodynia (Fig. 1C).

The location of tactile corpuscles at the apex of dermal papillae close to the skin surface is considered to endow these end organs with their exquisite sensitivity to skin deformation.^24^ These light touch detectors can be immunohistochemically identified with S100 for the lamellar Schwann cells that form a bulb structure, regarded to set tactile responsivity,^35^ that has close associations with the innervating myelinated afferent (identified with NF200) (Fig. 5Aa). To facilitate comparison of the innervation of incision-induced putative dermal papillae with those in the footpad, the density of S100+, S100+NF200+, S100+ bulb, and S100+ bulb with associated NF200+ nerve fibre containing dermal papillae were compared across PSD1-PSD7 (Fig. 5). A comparison with the control heel cannot be made because the dermal papillae structures do not exist in this region. A comparison with the control pad was made to enable comparison of the incision heel with the footpad structure that is specialized for tactile detection. The density of incision-induced putative dermal papillae containing S100+ immunostaining (examples in panel iv, Fig. 5Ab–d, f) and S100+NF200+ immunostaining (examples in panel iv Fig. 5Ad, f) was similar to the control pad across all postsurgical days (Fig. 5B, C). Interestingly, the density of putative dermal papillae containing an S100+ bulb (examples in panels iv, Fig. 5Ac, f), the lamellar cell wrappings regarded to set tactile responsivity,^35^ peaks and was comparable to the control pad at PSD2/3 (during the dynamic allodynia peak) but was significantly reduced at PSD7 (dynamic allodynia resolution) (Fig. 5D). Similarly, the density of putative dermal papillae containing an S100+ bulb, with an associated NF200+ nerve fibre (example in panel iv, Fig. 5Af), was comparable to the control pad at PSD3 but was significantly reduced at PSD7 (Fig. 5E). Quantification was conducted in tissue from male subjects, but the phenomenon of incision-induced tactile corpuscles was also observed in female subjects, as illustrated in Figure 5Af. Given that an S100+ bulb (irrespective of NF200) is used to identify tactile corpuscles,^34,35^ this measure was employed for the remainder of the study.

Figure 5.Hindpaw surgical incision-induced putative dermal papillae contain tactile corpuscles. (A) Immunostaining identification of NF200+ myelinated fibres (i) and S100+ lamellar cells (ii), overlay with Collagen IV (cyan) (iii), and higher magnification (iv) in control pad (a), PSD1 heel (b), PSD2 heel (c), PSD3 heel (d), and PSD7 heel (e). Representative a-e panels are from male tissue, whereas (f) is from female PSD2 tissue. Dashed line indicates incision side. Note that all (iv) panels with the exception of PSD7/eiv contain S100+ papillae. Arrows denote S100+ bulbs. Scale bar 100 μm (i–iii) and 50 μm (iv). Density of (B) S100+ dermal papillae and (C) S100+/NF200+ dermal papillae in incision heel and control pad across PSD. Density of (D) S100+ bulb containing dermal papillae and (E) S100+ bulb associated with NF200+ containing papillae reveals a peak at around PSD3, that is comparable to control pad, but is then reduced by PSD7. RM 2-way ANOVA, Tukey multiple comparisons posttests shown (*P* < 0.05*, *P* < 0.01**, *P* < 0.001***, *P* < 0.0001****). n = 6 to 8 male subjects per PSD.

**Figure:**
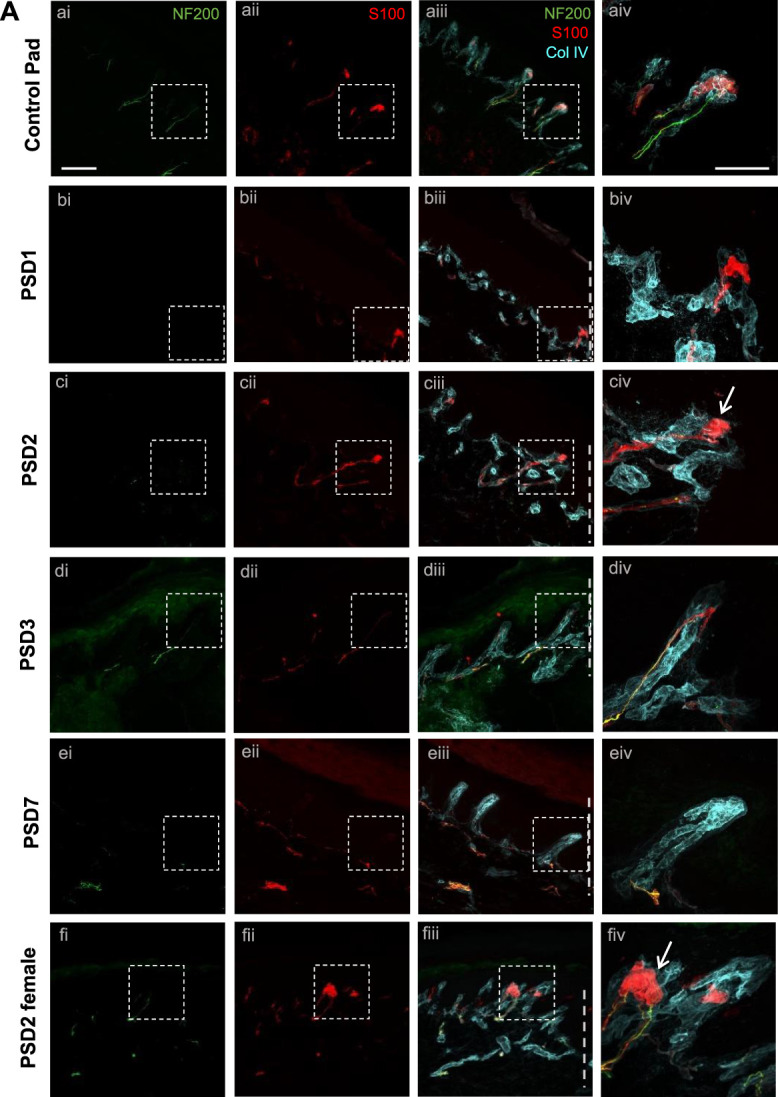

**Figure:**
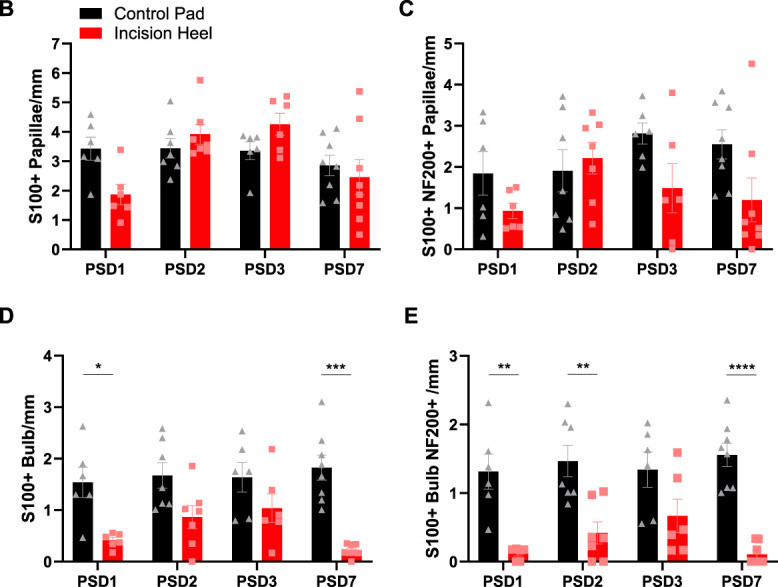

In summary, in the incision site surround, dermal papillae like structures form and are innervated with tactile corpuscles concurrent with dynamic allodynia.

### 3.4. Brain-derived neurotrophic factor-TrkB dependence

**Figure 6.**
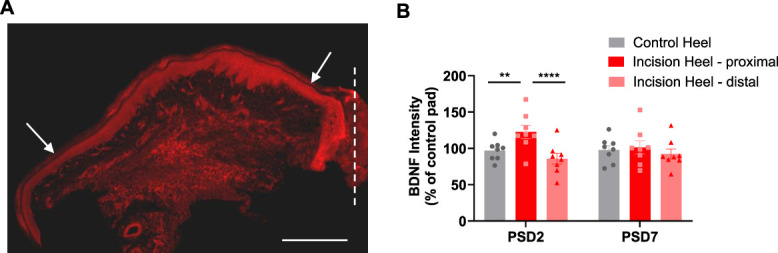
Hindpaw surgical incision-induced elevation of epidermal BDNF immunostaining. (A) BDNF immunostaining in PSD2 heel tissue. Dashed line indicates incision site. Arrows denote proximal and distal sites for intensity measures. Scale bar 500 μm. (B) BDNF immunostaining intensity, normalised to control pad, reveals an elevation proximal to the incision site on PSD2 that is resolved by PSD7. RM 2-way ANOVA followed by Tukey multiple comparisons posttests shown. (*P* < 0.01**, *P* < 0.0001****). N = 8 male (n = 4) and female (n = 4) subjects, per PSD.

Tactile corpuscles are innervated by TrkB expressing (TrkB+) afferents^14,35^ and require skin-derived BDNF^35^ and TrkB expression in primary sensory neurons^12,31^ for their development. Given that incision-induced tactile corpuscles appeared to be oriented towards the incision site, we assessed whether epidermal BDNF is increased close to the incision site. The intensity of BDNF immunofluorescence was greater close to the incision site compared with distal to the incision site or the control heel at PSD2 but not PSD7 (Fig. 6). These data suggest that incision increases BDNF levels in the adjacent epidermis, that induces the development of novel tactile corpuscles, that peaks ∼PSD2/3, and then, as BDNF levels normalise (PSD7), that innervation is retracted.

**Figure 7.**
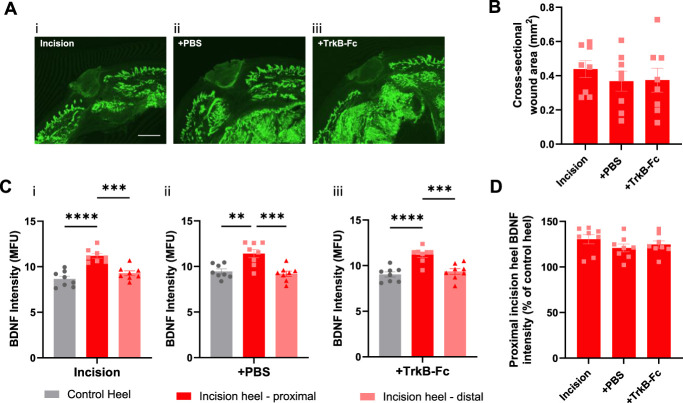
TrkB-Fc treatment does not impact wound cross-sectional area or incision-induced elevated BDNF immunostaining intensity. (A) Collagen IV immunostaining in PSD3 heel tissue from incision only (i), incision + vehicle (PBS) (ii), and incision + TrkB-Fc (iii) treatment groups. Scale bar 500 μm. (B) Cross-sectional wound area is not altered by treatment. (C) BDNF immunostaining intensity is elevated proximal to the incision site in PSD3 heel tissue from incision only (i), incision + vehicle (PBS) (ii), and incision + TrkB-Fc (iii) treatment groups. MFU, mean fluorescence units. (D) Elevated (normalised) BDNF immunostaining intensity proximal to the PSD3 heel incision site is not altered by treatment group. One-way ANOVA followed by Tukey multiple comparison tests shown (*P* < 0.01**, *P* < 0.001***, *P* < 0.0001****). n = 8 male subjects per group.

To test the BDNF-TrkB dependence of incision-induced tactile corpuscles, a TrkB-Fc chimera was administered locally to the incision surround on the day of surgery and also on PSD2. TrkB-Fc or vehicle treatment did not significantly alter the cross-sectional area of the wound, visualised in PSD3 tissue sections (Fig. 7A, B). The incision-induced elevation in epidermal BDNF immunostaining intensity on PSD3 was also not affected by treatment (Fig. 7C, D). Therefore, any impact of local TrkB-Fc chimera administration on innervation of the incision surround would not likely be secondary to changes in wound healing or epidermal BDNF levels.

**Figure 8.**
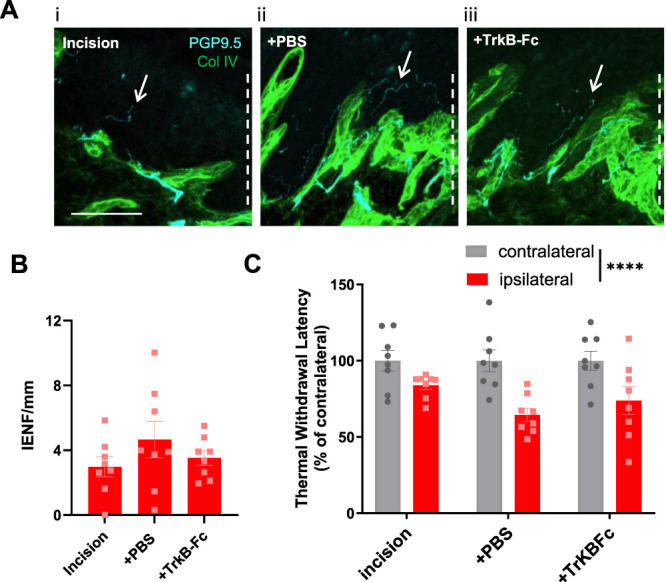
TrkB-Fc treatment does not impact IENFD or thermal hyperalgesia. (A) PSD3 heel tissue from incision only (i), incision + vehicle (ii), and incision + TrkB-Fc (c) treatment groups with PGP-9.5 immunostaining (cyan) and Collagen IV immunostaining (green). Dashed lines indicate the direction of incision site. Arrows denote PGP9.5 positive nerve fibres that cross the basement membrane into the epidermis (intraepidermal nerve fibres [IENFD]). Scale bar 100 µm. (B) IENFD in PSD3 incision heel is not altered by treatment group (one-way ANOVA) (C). Thermal hyperalgesia on PSD3 is not altered by treatment group (RM 2-way ANOVA, hindpaw *P* < 0.0001****; Sidak multiple comparison incision only vs TrKB-Fc ipsilateral hindpaw, *P* = 0.46). n = 8 male subjects per group.

**Figure 9.**
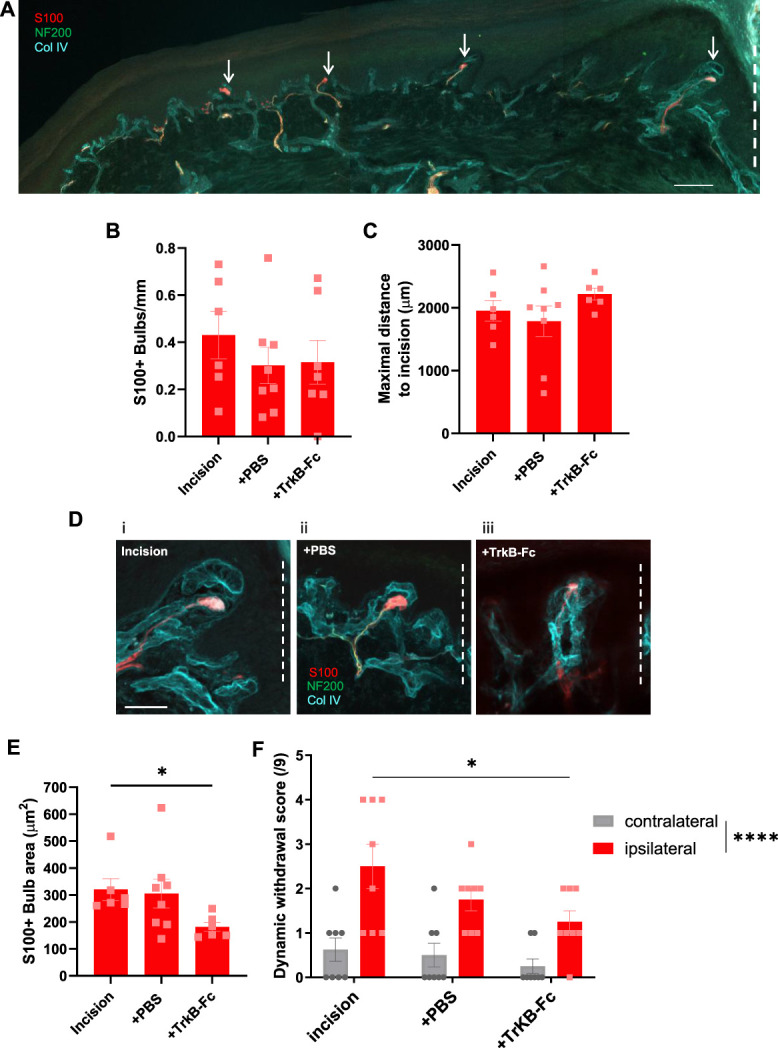
TrkB-Fc treatment decreases S100+ bulb area and dynamic allodynia. (A) Low magnification image of PSD3 heel tissue from incision only treatment group immunostained for NF200 (green), S100 (red), and Collagen IV (cyan) reveals novel tactile corpuscles (arrows) across the lateral extent of the heel. Dashed line indicates the direction of incision site. Scale bar 100 µm. (B) S100+ bulb density and (C). Maximal S100+ bulb distance from the incision site is not altered by treatment group. (D) Representative S100+ bulbs from PSD3 heel tissue from incision only (i), incision + vehicle (ii), and incision + TrkB-Fc (iii) treatment groups immunostained for NF200 (green), S100 (red), and Collagen IV (cyan). Dashed lines indicate the direction of incision site. Scale bar 50 µm. (E) TrkB-Fc treatment reduces S100+ bulb area of novel tactile corpuscles. Kruskal–Wallis test followed by Dunn multiple comparisons test. (F) TrkB-Fc treatment reduces incision-induced elevated dynamic withdrawal score. RM 2-way ANOVA followed by Sidak multiple comparisons. *P* < 0.05*, *P* < 0.0001****, n = 6 to 8 male subjects per group.

TrkB-Fc chimera should selectively impact the development of tactile corpuscles and not epidermal nociceptive innervation. Indeed, IENFD was unaltered between the treatment groups (Fig. 8A, B), and nociceptor-driven thermal hyperalgesia was not impacted by TrkB-Fc treatment (Fig. 8C). Figure 9A illustrates the lateral extent of incision-induced tactile corpuscles. TrkB-Fc treatment did not alter the density (Fig. 9B) of incision-induced tactile corpuscles or their maximal distance (Fig. 9C) from the incision site. Interestingly, the size of the S100+ bulb, the lamellar cell wrappings regarded to set tactile responsivity,^35^ was significantly reduced by approximately 50% in the TrkB-Fc group (Fig. 9D, E). Aligned with the TrkB-Fc reduction of tactile corpuscle size, dynamic allodynia was also significantly reduced by approximately 50%, as compared with the incision only group (Fig. 9F).

In summary, TrkB-Fc treatment significantly limited the development of tactile corpuscles and dynamic allodynia without affecting wound area, epidermal BDNF levels, nociceptive innervation, or thermal hyperalgesia.

## 4. Discussion

Surgical skin-muscle incision of the glabrous hindpaw resulted in a striking change to the structure and innervation of the skin. Putative dermal papillae developed oriented towards the incision site and contained novel tactile corpuscles, concurrent with behavioural evidence of dynamic allodynia. Increased BDNF levels surrounding the incision site alongside a reduction in both dynamic allodynia and tactile corpuscle size with TrkB-Fc treatment suggests that BDNF-TrkB–dependent novel tactile corpuscles are required for postsurgical tactile-evoked pain.

### 4.1. Skin structure and innervation

Postsurgical pain involves a neuropathic and inflammatory component.^18,28^ Therefore, the observed reduction in epidermal nociceptive innervation is supportive of a neuropathic component. This local nerve terminal damage will likely contribute, along with local inflammation, to peripheral sensitisation and provide an ongoing afferent drive that induces central sensitisation and unmasking of spinal allodynia circuits.^50^

We observed the development of putative dermal papillae in the incision site surround. In support of this observation, these structures are also evident but are not commented upon, in images from published rodent^6,53^ and pig incision^10,42^ studies. Rat skin is considered closer in structure to human skin than mouse skin.^22,48^ However, pig skin is most similar to human skin with a comparable epidermal thickness, vascular anatomy, collagen arrangement, and wound healing.^22,45,47^ Therefore, the observation of this phenomenon in rat skin and potentially also pig skin suggests that these findings may have translational relevance.

The observation of novel tactile corpuscles within the incision-induced putative dermal papillae, concurrent with dynamic allodynia, suggests that the novel tactile corpuscles contribute to dynamic allodynia. This proposition is supported by the reduction in both tactile corpuscle size and dynamic allodynia with TrkB-Fc treatment that blocks BDNF-TrkB signalling. Importantly, the novel tactile corpuscles may not exclusively be related to dynamic allodynia and may also contribute to punctate allodynia given the involvement of tactile corpuscles in neuropathic punctate allodynia.^14,21^ Moreover, it will be of interest to determine whether skin cutaneous incision is sufficient to elicit this phenomenon or whether a combined skin–muscle injury is required.^53^

### 4.2. Brain-derived neurotrophic factor-TrkB dependence

Tactile corpuscles require epidermal BDNF^35^ and TrkB receptor expression in primary sensory neurons to develop.^12,31^ Epidermal BDNF levels were elevated close to the incision site at PSD2/3 (Figs. 6, 7) when the novel tactile corpuscle density was maximal, suggesting a similar underlying mechanism. The novel innervation was reduced at the PSD7 time point presumably because epidermal BDNF levels had returned to baseline levels given that epidermal BDNF is required to maintain tactile corpuscle innervation.^37^

Local acute administration of TrkB-Fc resulted in approximately 50% reduction in S100+ bulb size. This is consistent with the finding of an increased S100+ bulb size in mice that overexpress BDNF in the epidermis.^30^ The S100+ bulb comprises the lamellar Schwann cells that are intimately associated with the innervating nerve fibres to comprise the tactile corpuscle. The innervating nerve fibres have multiple axonal protrusions that form adherens junctions with the Schwann cells that is thought to enable mechanical stimuli to stretch the axon at numerous sites, thereby activating proximal axonal Piezo2 mechanotransduction ion channels.^25^ Therefore, a smaller tactile corpuscle, with reduced Schwann cell nerve interactions, likely reflects a reduced tactile sensitivity, as observed in the footpad where smaller tactile corpuscles innervated with Ret-expressing afferents have a reduced tactile sensitivity as compared with the larger tactile corpuscles innervated with TrkB receptor-expressing afferents.^35^ Furthermore, in this study, the approximately 50% reduction in S100+ bulb size was accompanied by an approximately 50% reduction in tactile-evoked pain behaviours. Vehicle treatment, although not significant, appeared to cause a small reduction in tactile allodynia resulting in a nonsignificant difference from the incision + TrKB-Fc group. However, we speculate that this may result from the repeated injections locally irrigating the wound site and flushing out the inflammatory mediators that drive ongoing C-fibre activity that unmasks spinal allodynia circuits.^50^ Future TrKB-Fc studies should employ more refined administration strategies (topical,^27^ hydrogel^56^) and also include comparison with IgG controls to further validate the BDNF-TrKB dependence. Notably, TrkB-Fc treatment had no impact on thermal hyperalgesia that is mediated by NGF-TrkA signalling,^7,55^ suggesting complementary neurotrophin signalling for mechanical vs thermal hypersensitivity postsurgery.

TrkB-Fc treatment did not impact the innervation density of the novel tactile corpuscles potentially reflecting limitations of the administration approach. The PSD0 time point may have had limited impact if drug washout occurred before an increase in epidermal BDNF levels. Furthermore, at the PSD2 time point, the tactile corpuscles may already be forming within the putative papillae. Therefore, the fact that this acute administration had a concomitant impact on both tactile corpuscle size and dynamic allodynia holds promise for more effective administration strategies. Slow-release approaches using topical^27^ or injectable^56^ hydrogel systems could provide ongoing blockade of BDNF-TrkB signalling and improved efficacy. Moreover, a local peripheral treatment that avoids the side effects of CNS targeted agents could be a promising strategy for postsurgical tactile pain. However, it is essential to confirm that any treatment regimen does not negatively impact wound healing.^46^ Of note, acute administration of TrkB-Fc did not impact wound cross-sectional area, suggesting that it does not interfere with wound healing. Further evidence that supports targeting of the BDNF-TrkB system for postsurgical tactile pain is identification of a genetic variant of BDNF that decreases patient risk of chronic postsurgical pain and the observation that mice with this BDNF polymorphism have reduced mechanical allodynia following hindpaw plantar incision.^49^

### 4.3. Acute vs chronic postsurgical pain

The plantar hindpaw-incision model is considered to model acute rather than persistent postsurgical pain.^18^ However, acute postsurgical pain is a significant risk factor for the development of persistent postsurgical pain following multiple types of surgery.^4,18,28,33^ Given the challenges regarding the use of opioids for the management of acute postsurgical pain,^3,32^ including potential facilitation of persistent postsurgical pain,^18^ the identification of novel underlying acute postsurgical pain mechanisms with therapeutic potential, like the current observation, are required. This could enable development of preventative strategies to treat acute pain and thereby limit the development of persistent postsurgical pain.^11^

In summary, this discovery of novel tactile corpuscles in the incision site surround has identified a potential therapeutically tractable mechanism that could limit the burden of postoperative pain.

## Disclosures

The authors have no conflict of interest to declare.
